# Prenatal ultrasound phenotypic and genetic etiology of the 17q12 microduplication syndrome

**DOI:** 10.3389/fped.2022.910497

**Published:** 2022-08-10

**Authors:** Meiying Cai, Min Lin, Nan Guo, Meimei Fu, Liangpu Xu, Na Lin, Hailong Huang

**Affiliations:** Medical Genetic Diagnosis and Therapy Center, Fujian Maternity and Child Health Hospital College of Clinical Medicine for Obstetrics and Gynecology and Pediatrics, Fujian Key Laboratory for Prenatal Diagnosis and Birth Defect, Fujian Medical University, Fuzhou, China

**Keywords:** 17q12 microduplication syndrome, fetal ultrasound phenotype, pregnancy outcome, chromosome microarray analysis, duodenal obstruction

## Abstract

**Background:**

Several studies have reported on the clinical phenotype of the 17q12 microduplication syndrome, a rare autosomal dominant genetic disorder, in children and adults, but few have reported on its prenatal diagnosis. This study analyzed the prenatal ultrasound phenotypes of the 17q12 microduplication syndrome to improve the understanding, diagnosis, and monitoring of this disease in fetuses.

**Methods:**

A retrospective analysis of 8,200 pregnant women who had received an invasive antenatal diagnosis at tertiary referral hospitals between January 2016 and August 2021 was performed. Amniotic fluid or cord blood was sampled from the pregnant women for karyotyping and chromosome microarray analysis (CMA).

**Results:**

The CMA revealed microduplication in the 17q12 region of the genome in five fetuses, involving fragments of about 1.5–1.9 Mb. Five fetuses with the 17q12 microduplication syndrome had different prenatal ultrasound phenotypes, including duodenal obstruction (two fetuses); mild ventriculomegaly, dysplasia of the septum pellucidum, agenesis of the corpus callosum (one fetus); and a strong echo in the left ventricle only (one fetus). The ultrasound phenotype of one fetus was normal. Among the five fetuses with the 17q12 microduplication syndrome, the parents of three refused CNV segregation analysis, while CNV segregation analysis was performed for the remaining two fetuses to confirm whether the disorder was inherited maternally or paternally, with normal phenotypes. After genetic counseling, the parents of those two fetuses chose to terminate the pregnancy, while the parents of the three unverified fetuses continued the pregnancy, with normal follow-up after birth.

**Conclusion:**

Although prenatal ultrasound phenotypes in fetuses with the 17q12 microduplication syndrome are highly variable, our study has highlighted the distinct association between duodenal obstruction and the 17q12 microduplication syndrome. Understanding the relationship between the pathogenesis of the 17q12 microduplication in prenatal ultrasound phenotypes and its long-term prognosis will contribute to better genetic counseling concerning the 17q12 microduplication syndrome, which is still a challenge.

## Introduction

The 17q12 microduplication syndrome is a rare autosomal dominant genetic disease, and involves the occurrence of a 1.4 Mb microduplication at the 17q12 region of the chromosome, which contains pathogenic genes such as *HNFIB, LHXI*, and *ACACA* (1). The primary clinical manifestations of 17q12 microduplication syndrome are developmental delay, abnormal brain development, epilepsy, cognitive impairment, behavioral abnormalities, esophageal atresia, abnormal eye development, cardiac abnormality, and sexual reversal (2–7). However, unlike the clinical phenotypes of the 17q12 microduplication syndrome in adults and children (8), the ultrasonic phenotypes in fetuses have rarely been reported.

Microdeletions and micro duplications are common chromosomal abnormalities. However, the standard diagnostic method inclinical cytogenetics, the G-banding karyotype analysis, can usually only detect changes greater than 5 Mb. In contrast, the chromosome microarray analysis (CMA) can be used for genome-wide copy number analysis, which can quickly and effectively detect chromosomal imbalance variations from conventional karyotype analyses, as well as microdeletions and microduplications throughout the genome. Compared to that of karyotype analysis, the advantages of CMA include high resolution, high sensitivity, accuracy, and easy automation (9). Consequently, the widespread use of CMA in clinical and research laboratories has led to the rapid discovery of chromosomal microdeletions and microduplications associated with various diseases (10, 11). In this study, a combination of karyotype analysis and CMA was used to examine 8,200 pregnant women who had received an antenatal diagnosis at tertiary referral hospitals between January 2016 and August 2021. Clinical diagnosis, genetic counseling, and treatment were provided based on the analysis of the prenatal ultrasonic phenotype of five fetuses diagnosed with the 17q12 microduplication syndrome.

## Materials and methods

### Subjects

A retrospective analysis of 8,200 pregnant women who underwent invasive antenatal diagnoses for karyotype analysis and CMA at tertiary referral hospitals between January 2016 and August 2021 was performed. The mean age of the pregnant women was 29.1 years (range: 18–49 years), and the mean gestational age was 24.3 weeks (range: 16–38 weeks). Amniotic fluid or cord blood was extracted depending on the gestational age. All pregnant women received genetic counseling and signed informed consent prior to the invasive diagnosis. This study was approved by the Medical Ethics Committee of Fujian Maternal and Child Health Hospital (no. 2014042).

### Karyotype analysis

Amniocentesis was performed to extract 30 mL of amniotic fluid under ultrasound localization, or 2 mL of cord blood was sampled from the umbilical cord. Amniotic fluid samples or umbilical cord blood samples were extracted and inoculated in a 1,640 medium (Bosheng Company) for culturing. The umbilical cord blood samples were cultured for 3 days, then the cells were harvested. After 8 days of amniotic fluid culture, cell morphology and exchange fluid were assessed. Colchicine was added to the cells exhibiting good morphology in the growth phase, to accelerate the cell growth to the metaphase of the mitotic cycle. Then cell harvest, drop tablets, and Giemsa dye banding were carried out. Finally, karyotype collection was carried out using the GSL-120 automatic chromosome scanning platform, and karyotype calculation and analysis were performed. Karyotypes were named according to ISCN 2016; 40 karyotypes were counted in each case, and 5 were analyzed, while another 20 karyotypes were counted and analyzed in case of an abnormality.

### Chromosome microarray analysis

Genomic DNA was extracted using the QIAamp DNA Blood Mini Kit. Genomic DNA digestion, PCR amplification, product purification, fragmentation, labeling, hybridization, washing, and scanning were performed according to the standard operating procedures listed by the manufacturer. The Chromosome Analysis Suite (ChAS) V3.2 software (Affymetrix, California, United States) was used for data analysis, and the CMA results were analyzed using related databases to determine the properties of chromosome copy number variations (CNVs). Multiple online public databases were referred, such as Database of Genomic Variants,^1^ DECIPHER Database,^2^ Online Mendelian Inheritance in Man,^3^ the International Standards for Cytogenomic Arrays,^4^ PubMed,^5^ and ClinGen.^6^ According to the American Medical Genetics Guide (12), CNVs can be divided into five categories as follows: pathogenic, likely pathogenic, copy number variation of uncertain clinical significance (VUS), likely benign, and benign. For fetuses with VUS, it is recommended that the respective parents undergo the CMA test on peripheral blood samples, combined with pedigree analysis, to further clarify the nature of the CNVs.

### Pregnancy outcomes and follow-up

All cases were followed up *via* telephonic communication, to gather information on fetal development, pregnancy outcome, and postpartum growth and development.

## Results

### Ultrasonic phenotype of the fetus

**FIGURE 1:**
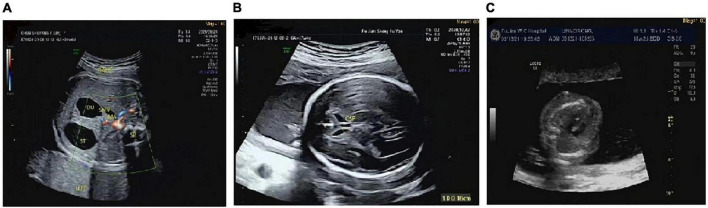
The prenatal ultrasound phenotype of the fetuses. **(A)** Showed duodenal obstruction. **(B)** Showed mild ventriculomegaly, dysplasia of the septum pellucidum, agenesis of the corpus callosum. **(C)** Showed a strong echo in the left ventricle.

There were differences in the prenatal ultrasound phenotypes of the five fetuses with the 17q12 microduplication syndrome. The typical prenatal phenotype of 17q12 microduplication syndrome was duodenal obstruction. The frequency of dudodenal obstruction in patients with17q12 duplication (40.0%, 2/5) was significantly higher than in patients without 17q12 duplication (0.2%, 17/8,195) in our study. We observed duodenal obstruction in two fetuses; mild ventriculomegaly, dysplasia of the septum pellucidum, agenesis of the corpus callosum in one fetus; and a strong echo in the left ventricle in only one fetus. Although the ultrasound phenotype of one fetus was normal, the prenatal diagnosis of this fetus indicated a high-risk for Down’s syndrome. The ultrasonic phenotypes of these five fetuses are shown in Table 1 and Figure 1.

**TABLE 1:** Ultrasonic phenotype, CMA results, and pregnancy outcome in 5 fetuses with the 17q12 microduplication syndrome.

Case	GA	CMA results	Size (Mb)	Ultrasonic phenotype	Inheritance	Pregnancy outcome
P6118	18^+^2	arr[hg19] 17q12 (34,440,088–36,351,919) × 3	1.9	Strong echo in left ventricle	Paternal	Normal physical and mental development
P9430	26^+^2	arr[hg19] 17q12 (34,822,465–36,378,678) × 3	1.5	Mild ventriculomegaly, dysplasia of the septum pellucidum, and agenesis of the corpus callosum	Maternal	Normal physical and mental development
R2938	18	arr[hg19] 17q12 (34,440,088–36,243,365) × 3	1.8	Normal phenotype, high-risk for Down’s screening	−	TP
R2995	23^+^6	arr[GRCh37] 17q12 (34,440,088–36,243,365) × 3	1.8	Duodenal obstruction	−	TP
R3181	28^+^4	arr[GRCh37] 17q12 (34,426,244–36,300,630) × 3	1.8	Duodenal obstruction	−	Duodenal surgery

GA, gestational age; TP, termination of pregnancy.

### Karyotype analysis

The karyotype analyses of the five cases were negative.

### Chromosome microarray analysis

**FIGURE 2:**
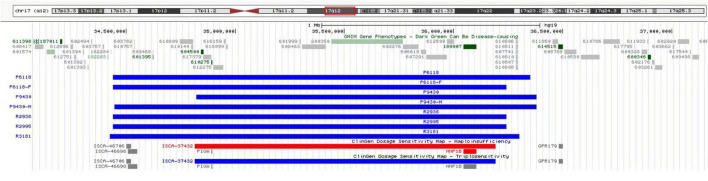
CMA revealed microduplication in the 17q12 region of the genome in five fetuses, involving fragment size about 1.5–1.9 Mb, including *HNF1B* (189907), *LHX1* (601999), *CCL4L1* (603782), *CCL4L2* (610757), *CCL3L1* (601395), and 15 other *OMIM* genes.

Of the 8,200 fetuses subjected to CMA, duplication of the 17q12 region of the genome was detected in five fetuses. The involved fragment size was about 1.5–1.9 Mb and included *HNF1B* (189907), *LHX1* (601999), *CCL4L1* (603782), *CCL4L2* (610757), *CCL3L1* (601395), and 15 other *OMIM* genes (Figure 2).

### Chromosome microarray analysis and pedigree analysis of the families

Parents of three fetuses (R2938, R2995, and R3181) refused CNV segregation analysis. The parents of the other two fetuses (P6118 and P9430) agreed to pedigree analysis, and 17q12 microduplication syndrome was verified. The pedigree analysis of case P6118 confirmed that the microduplication of 17q12 was received from the father with abnormal phenotype. In the case of P9430, the CNV segregation analysis confirmed that the microduplication of 17q12 was derived from the mother with a normal phenotype.

### Pathogenicity analysis

The literature review and database analysis showed that the triple dose sensitivity score of the 17q12 microduplication region was 3 in the ClinGen database, and the reported penetrance was about 21.1% (PMID: 23258348). The clinical phenotypes of patients vary greatly and may manifestas intellectual disability, microcephaly, epilepsy, brain abnormalities, esophageal atresia, urinary malformation, and other abnormalities; although some patients may not have any distinct clinical abnormalities. This genome copy number duplication is a pathogenic variation, and due to the existence of genetic heterogeneity, there are incomplete phenotypes exhibiting different manifestations.

### Pregnancy outcome

The parents of two fetuses (R2938 and R2995) refused CNV segregation analysis and chose to terminate the pregnancy after being informed of the possible risks through genetic counseling. Two cases (P6118 and P9430) were confirmed by pedigree analysis to inherit the 17q12 microduplication from a parent with a normal phenotype, and the fetuses were followed up with till approximately 3 years of age after birth, exhibiting normal physical, and mental development. The parents of case R3181 refused CNV segregation analysis, but chose to continue the pregnancy. The ultrasound phenotype of the fetus after birth was consistent with the phenotype exhibiting duodenal obstruction, so neonatal duodenal surgery was performed. After the surgery, at 4 months of age, the baby exhibited normal physical, and mental development.

## Discussion

The recombination of the 17q12 region leads to a change in the dosage of the sensitive gene leading to a microdeletion or a microduplication of the chromosome (3). The 17q12 microdeletion is common and the clinical phenotype is also clear (13, 14). Microdeletions of the chromosome at the 17q12 region often presents in patients asrenal cysts, maturity-onset diabetes of the young type 5, and impaired cognitive development of the nervous system (15). Microduplication of the 17q12 region is a rare microduplication syndrome with variable clinical phenotypes (16). Most of the patients with the 17q12 microduplication syndrome report neurological and psychiatric symptoms, including hypophrenia, learning difficulties, behavioral problems, and epilepsy (1, 17). Congenital heart disease, duodenal atresia, growth abnormalities, microcephaly, renal abnormalities, and ophthalmic abnormalities have also been reported in some adults, but are not identical (1, 4, 16–20). All of these are clinical phenotypes reported in adults and children; however, the ultrasonic phenotypes of fetuses with the 17q12 microduplication syndrome are rarely reported.

This study summarizes the reported ultrasound phenotypes of fetuses with the 17q12 microduplication syndrome (Table 2). Lu et al. reported that the 17q12 microduplication was associated with congenital heart defects (21). However, no ultrasound phenotype of congenital heart defects was found in our study. The typical prenatal phenotype of 17q12 microduplication syndrome was duodenal obstruction. Zhou et al., Zhang et al., and Zhang et al. reported that the ultrasound phenotype in a fetus with the 17q12 microduplication syndrome was a “Double Bubble” sign (22–24). The “Double Bubble” sign is an ultrasound characteristic representing duodenal obstruction. The ultrasound phenotype of duodenal obstruction was also detected in two fetuses in our study, and was the most common ultrasound phenotype observed in our study. We found the frequency of dudodenal obstruction in patients with 17q12 duplication (40.0%, 2/5) was significantly higher than in patients without 17q12 duplication (0.2%, 17/8,195) in our study. Hence, a fetal ultrasound phenotype of duodenal obstruction is potentially indicative of the occurrence of the 17q12 microduplication syndrome in the fetus. When the prenatal ultrasound phenotype of fetuse is duodenal obstruction, 17q12 microduplication syndrome should be highly suspected. Li et al. also reported mild ventriculomegaly, microcephaly, and agenesis of the corpus callosum in the ultrasound phenotype of a fetus with the 17q12 microduplication syndrome (25). In our study, a fetal ultrasound phenotype was also found to present mild ventriculomegaly, dysplasia of the septum pellucidum, and agenesis of the corpus callosum. These data suggest that mild ventriculomegaly and agenesis of the corpus callosum in the fetal ultrasound phenotype may also be indicators of the 17q12 microduplication syndrome. In contrast, Chen et al. had reported the absence of obvious ultrasound abnormalities in a fetus with the 17q12 microduplication syndrome (26). This is in accordance to the normal ultrasound phenotype we observed in our study. In addition, we also observed a strong echo in only the left ventricle in the ultrasound phenotype of a fetus in our study, which has not been reported previously. The fetal ultrasound phenotypes reported in our study enrich the clinical database, given that the clinical phenotypes of the 17q12 microduplication syndrome are complex and diverse, requiring large sample data and more in-depth clinical studies.

**TABLE 2:** Frequency of phenotypes in current study and previously published cases.

Phenotypes	Total number in reported cases (with phenotype/total of cases)	Current cases (with phenotype/reporting)	References
Duodenal atresia	6/47	2/5	(1, 22, 23, 24)
Polyhydramnios	4/47	0/5	(3, 22, 23, 27)
Pulmonary artery stenosis	1/47	0/5	(22)
Tricuspid regurgitation	1/47	0/5	(22)
FL and HL < 5 centile	1/47	0/5	(22)
BPD and HC < 5 centile	1/47	0/5	(22)
Tracheoesophageal fistula	2/47	0/5	(3, 32)
Anal atresia	1/47	0/5	(32)
Congenital heart defects	1/47	0/5	(21)
Lateral ventricle broadening	1/47	0/5	(25)
Esophagus abnormalities	3/47	0/5	(1)
Placental abruption	1/47	0/5	(4)
IUGR	1/47	0/5	(36)
Strong echo in left ventricle	1/47	1/5	–
Mild ventriculomegaly	1/47	1/5	–
Dysplasia of the septum pellucidum	1/47	1/5	–
Agenesis of the corpus callosum	2/47	1/5	(25)

BPD, biparietal diameter; FL, femur length; HL, humerus length; IUGR, intrauterine growth restriction.

In this study, the microduplication region was found to contain 15 *OMIM* genes, including *HNF1B* (189907), *LHX1* (601999), *CCL4L1* (603782), *CCL4L2* (610757), and *CCL3L1* (601395), among which *HNF1B* is the most closely related to the disease (26–31). The *HNF1B* gene is mostly associated with kidney disorders, followed by neurodevelopmental disorders (18, 32–34). The *HNF1B* gene is expressed in the embryo in organs such as the kidney and the liver, and its heterozygous point mutations and small deletions cause renal cysts and juvenile or adult diabetes. However, none of the five fetuses with the 17q12 microduplication syndrome included in this study showed abnormal kidney development. The protein encoded by the *LHX1* gene is an important regulator of the formation of the kidney and the urogenital system, and is also related to the development of the nervous system, playing a significant role in the growth and development and structural stability of the axons innerve cells (17, 35, 36). In this study, a fetus with the 17q12 microduplication syndrome displayed conditions consistent with the findings of previous literature, and its ultrasound phenotype showed mild ventriculomegaly and agenesis of the corpus callosum. At present, the functions of all genes contained in the 17q12 region and their connection to the phenotypes are not clear; hence, further studies should be conducted.

Rosenfeld et al. found that the penetrance rate of clinical manifestations in patients with the 17q12 microduplication syndrome was 21.1% (8). This incomplete penetrance and variable expression, possibly involving phenotypes with neurodevelopmental disorders, presents challenges to prenatal counseling and phenotype prediction after birth. The 17q12 microduplication syndrome is 90% inherited by autosomal dominance from the least affected parent or the parent with a normal phenotype (26). The microduplication of the 17q12 region in two fetuses in this study was pedigree verified to be inherited from a parent with a normal phenotype. In both cases, the parents chose to continue the pregnancy after being fully informed that the 17q12 microduplication syndrome had a broad phenotype and incomplete penetrance. We followed up with the two children till approximately 3 years after birth; their physical and mental development was normal. This suggests that it is necessary to conduct CNV segregation analysis for the detection of 17q12 microduplication syndrome. CNV segregation analysis can help detect sources of mutation and understand the mechanism of chromosomal mutation. It can also help develop a preliminary phenotype estimate and diagnose mild illnesses after birth, although follow-up and treatment are highly recommended. Among the cases without parental CNV segregation analysis, two chose termination of pregnancy, and one chose continuation of pregnancy. The intrauterine ultrasound phenotype of the continued pregnancy consisted of duodenal obstruction and mild tricuspid regurgitation. The clinical phenotype of the newborn was duodenal obstruction. After surgical treatment, we followed up with the newborn for up to 4 months. The physical and mental development of the newborn was normal. This suggests that a good fetal pregnancy outcome can be expected despite the fetus having the17q12 microduplication syndrome. However, neuropsychiatric assessment and monitoring should be conducted in the future, throughout childhood and adulthood, for the three cases showing normal physical and mental development after birth. The limitations of this study are its flaws which could be the result of unavailability of resources, and small sample size. However, this data would help validate the possibility of a good fetal pregnancy outcome despite a 17q12 microduplication syndrome diagnosis in the fetus.

## Conclusion

In conclusion, the 17q12 microduplication syndrome can be associated with variable phenotypes. Our study on the ultrasonic phenotype of fetuses with the 17q12 microduplication syndrome is broadly consistent with previous studies; however, our study also highlights the close correlation between the ultrasonic phenotype of fetuses with duodenal obstruction and the 17q12 microduplication syndrome. Genetic counseling for the 17q12 microduplication syndrome remains a challenge for obstetricians, parents, genetic counselors, and clinicians. Further research on the relationship between the pathogenesis of fetal ultrasound phenotype with the 17q12 microduplication syndrome and its long-term prognosis would be helpful in improving genetic counseling for the 17q12 microduplication syndrome.

## Data availability statement

The data presented in this study are deposited in the Gene Expression Omnibus repository, accession number: acc=GSE208291, https://www.ncbi.nlm.nih.gov/geo/query/acc.cgi?acc=GSE208291.

## Ethics statement

The studies involving human participants were reviewed and approved by the Medical Ethics Committee of Fujian Maternal and Child Health Hospital. The patients/participants provided their written informed consent to participate in this study.

## Author contributions

MC wrote the manuscript. NG collected and interpreted the data. MF managed the study. LX and ML designed the study. NL and HH revised the manuscript. All authors contributed to the article and approved the submitted version.

## Footnotes

1
http://projects.tcag.ca/variation/

2
http://decipher.sanger.ac.uk/

3
http://www.omim.org

4
http://www.iscaconsortium.org/

5
http://www.ncbi.nlm.nih.gov/pubmed/

6
http://www.clinicalgenome.org/
